# Supplementary material for: A UMLS-based spell checker for natural language processing in vaccine safety
Source: BMC Med Inform Decis Mak. 2007 Feb 12;7:3. doi: 10.1186/1472-6947-7-3 (PMC1805499; doi:10.1186/1472-6947-7-3)
Supplement: Additional file 13 — RAP application directory. Sets up directory for RAP (RDF application for PHP) source code [file 1472-6947-7-3-S13.gz › rap/doc/phpdoc/__filesource/fsource_syntax__syntaxRdfSerializer.php.html]

xml version="1.0" encoding="iso-8859-1"?


File Source for RdfSerializer.php


# Source for file RdfSerializer.php

Documentation is available at RdfSerializer.php

```
1. <?php


4. // ----------------------------------------------------------------------------------
5. // Class: RdfSerializer
6. // ----------------------------------------------------------------------------------


10. /**
11. * An RDF seralizer.
12. * Seralizes models to RDF syntax. It supports the xml:base, xml:lang, rdf:datatype and
13. * rdf:nodeID directive.
14. * You can choose between different output syntaxes by using the configuration methods
15. * or changing the configuration default values in constants.php.
16. * This class is based on the java class edu.unika.aifb.rdf.api.syntax.RDFSerializer by Boris Motik.
17. *
18. * <BR><BR>History:<UL>
19. * <LI>12-14-2004                 : bug in function serialize() fixed by S�ren Auer(auer@informatik.uni-leipzig.de) </LI>
20. * <LI>12-06-2004                 : improved namespace handling added (tobias.gauss@web.de)</LI>
21. * <LI>01-06-2004                 : Empty model bug fixed.</LI>
22. * <LI>03-25-2004                 : Bug in saveAs() fixed by inkel(inkel-php@f14web.com.ar)</LI>
23. * <LI>03-21-2004                 : Support for xml default namespace added
24. *                                   Methods changed: serialize(), writeNamespaceDeclarations(), getElementText()</LI>
25. * <LI>11-17-2003                : Support for XMLLiterals added.</LI>
26. * <LI>07-27-2003                : Functions addapted to the new class tree (MemModel extends Model)</LI>
27. * <LI>07-24-2003                : Bug in writeAbsoluteResourceReference() fixed by Paul Cowles(paul@semaview.com) </LI>
28. * <LI>02-21-2003                : saveAs method added.</LI>
29. * <LI>02-12-2003                : rdf:type and bNode reference bug fixed.</LI>
30. * <LI>01-15-2003                : pass-by-reference bug fixed.</LI>
31. * <LI>01-10-2003                : rdf:datatype and $useAttributes bug fixed.</LI>
32. * <LI>12-04-2002                : Added support for rdf:datatype (writeContentStatements())</LI>
33. * <LI>12-04-2002                : Added rdf:nodeID attribute for blank nodes (writeSubjectURI, writeResourceReference)</LI>
34. * <LI>10-03-2002                : Bug in collectNamespaces() fixed.</LI>
35. * <LI>09-15-2002                : First version of this class.</LI>
36. * </UL>
37. *
38. * @version  V0.9.1
39. * @author Chris Bizer <chris@bizer.de>, Boris Motik <motik@fzi.de>, Daniel Westphal <dawe@gmx.de>, Leandro Mariano Lopez <llopez@xinergiaargentina.com>
40. *
41. * @package syntax
42. * @access  public
43. *
44. */
45. class RdfSerializer extends Object {

47. // configuration

49. var $use_entities;
50. var $use_attributes;
51. var $sort_model;
52. var $rdf_qnames;
53. var $use_xml_declaration;

55. // properties

57. var  $m_defaultNamespaces  = array();
58. var  $m_namespaces = array();
59. var  $m_nextAutomaticPrefixIndex;
60. var  $m_out;
61. var  $m_baseURI;
62. var  $m_statements = array();
63. var  $m_currentSubject;
64. var  $m_rdfIDElementText;
65. var  $m_rdfAboutElementText;
66. var  $m_rdfResourceElementText;
67. var  $m_groupTypeStatement;
68. var  $m_attributeStatements = array();
69. var  $m_contentStatements = array();
70. var  $rdf_qname_prefix;

72. /**
73. * Constructor
74. *
75. * @access   public
76. */
77. function RdfSerializer() {

79. // default serializer configuration
80. $this->use_entities = SER_USE_ENTITIES;
81. $this->use_attributes = SER_USE_ATTRIBUTES;
82. $this->sort_model = SER_SORT_MODEL;
83. $this->rdf_qnames = SER_RDF_QNAMES;
84. $this->use_xml_declaration = SER_XML_DECLARATION;

86. global $default_prefixes;
87. foreach($default_prefixes as $key => $value){
88. $this->addNamespacePrefix($key,$value);
89. }

91. require_once(RDFAPI_INCLUDE_DIR.PACKAGE_UTILITY);
92. }

94. /**
95. * Serializer congiguration: Sort Model
96. * Flag if the serializer should sort the model by subject before serializing.
97. * TRUE makes the RDF code more compact.
98. * TRUE is default. Default can be changed in constants.php.
99. *
100. * @param     boolean
101. * @access    public
102. */
103. function configSortModel($bool) {
104. $this->sort_model = $bool;
105. }

107. /**
108. * Serializer congiguration: Use Entities
109. * Flag if the serializer should use entities for URIs.
110. * TRUE makes the RDF code more compact.
111. * FALSE is default. Default can be changed in constants.php.
112. *
113. * @param     boolean
114. * @access    public
115. */
116. function configUseEntities($bool) {
117. $this->use_entities = $bool;
118. }

120. /**
121. * Serializer congiguration: Use Attributes
122. * Flag if the serializer should serialize triples as XML attributes where possible.
123. * TRUE makes the RDF code more compact.
124. * FALSE is default. Default can be changed in constants.php.
125. *
126. * @param     boolean
127. * @access    public
128. */
129. function configUseAttributes($bool) {
130. $this->use_attributes = $bool;
131. }

133. /**
134. * Serializer congiguration: Use Qnames
135. * Flag if the serializer should use qualified names for RDF reserved words.
136. * TRUE makes the RDF code more compact.
137. * TRUE is default. Default can be changed in constants.php.
138. *
139. * @param     boolean
140. * @access    public
141. */
142. function configUseQnames($bool) {
143. $this->rdf_qnames = $bool;
144. }

146. /**
147. * Serializer congiguration: Use XML Declaration
148. * Flag if the serializer should start documents with the xml declaration
149. * <?xml version="1.0" encoding="UTF-8" ?>.
150. * TRUE is default. Default can be changed in constants.php.
151. *
152. * @param             boolean
153. * @access    public
154. */
155. function configUseXmlDeclaration($bool) {
156. $this->use_xml_declaration = $bool;
157. }


160. /**
161. * Adds a new prefix/namespace combination.
162. *
163. * @param     String $prefix
164. * @param     String $namespace
165. * @access    public
166. */
167. function addNamespacePrefix($prefix, $namespace) {
168. $this->m_defaultNamespaces[$prefix] = $namespace;
169. }

171. /**
172. * Serializes a model to RDF syntax.
173. * RDF syntax can be changed by config_use_attributes($boolean), config_use_entities($boolean),
174. * config_sort_model($boolean).
175. * NOTE: There is only one default namespace allowed within an XML document.
176. *       Therefore if SER_RDF_QNAMES in constants.php is set to FALSE and you pass
177. *       another $xml_default_namespace as parameter, the model will be serialized
178. *       as if SER_RDF_QNAMES were set to TRUE.
179. *
180. * @param     object MemModel $model
181. * @param     String $encoding
182. * @return    string
183. * @access    public
184. */
185. function & serialize(&$model, $xml_default_namespace = NULL, $encoding = DEFAULT_ENCODING) {

187. if ($xml_default_namespace) {

189. if ($xml_default_namespace == RDF_NAMESPACE_URI) {
190. $this->rdf_qnames = FALSE;
191. unset($this->m_defaultNamespaces[RDF_NAMESPACE_PREFIX]);
192. }
193. elseif ($xml_default_namespace == RDF_SCHEMA_URI) {
194. unset($this->m_defaultNamespaces[RDF_SCHEMA_PREFIX]);
195. }
196. elseif (!SER_RDF_QNAMES)
197. $this->rdf_qnames = TRUE;

199. $this->addNamespacePrefix(NULL, $xml_default_namespace);
200. }

202. //copy parsed namespaces
203. $nsps = array();
204. $nsps = $model->getParsedNamespaces();
205. foreach($this->m_defaultNamespaces as $prefix => $namespace){
206. if(!isset ($nsps[$namespace]))
207. $nsps[$namespace] = $prefix;
208. }

210. // define rdf prefix (qname or not)
211. if ($this->rdf_qnames){
212. if(isset($nsps[RDF_NAMESPACE_URI])){
213. $this->rdf_qname_prefix = $nsps[RDF_NAMESPACE_URI].':';
214. }else{
215. $this->rdf_qname_prefix = RDF_NAMESPACE_PREFIX . ':';
216. }

218. }else{
219. $this->rdf_qname_prefix = '';
220. }
221. // check if model is empty
222. if ($model->size() == 0) return "<". $this->rdf_qname_prefix . RDF_RDF ." xmlns:rdf='".RDF_NAMESPACE_URI."' />";

224. foreach($nsps as $ns => $pre){
225. $this->m_namespaces[$pre] = $ns;
226. }


229. // set base URI
230. if ($model->getBaseURI()==NULL)
231. $this->m_baseURI="opaque:uri";
232. else
233. $this->m_baseURI=$model->getBaseURI();


236. if ($this->sort_model) {
237. // sort the array of statements

239. foreach($model->triples as $key => $statement) {
240. $stmkey = $statement->subj->getURI() .
241. $statement->pred->getURI() .
242. (is_a($statement->obj,'Literal')?'"'.$statement->obj->getLabel().'"@'.$statement->obj->getLanguage().'^^'.$statement->obj->getDatatype():$statement->obj->getURI());
243. $this->m_statements[$stmkey] = $statement;
244. }
245. ksort($this->m_statements);

247. /*
248. // Sort using the PHP usort() function. Slower :-(
249. $this->m_statements = $model->triples;
250. usort($this->m_statements, "statementsorter");
251. */
252. } else {
253. $this->m_statements = $model->triples;
254. }
255. // collects namespaces
256. $this->m_nextAutomaticPrefixIndex=0;
257. $this->collectNamespaces($model);

259. // start writing the contents
260. $this->m_out="";
261. if ($this->use_xml_declaration)
262. $this->m_out .= '<?xml version="1.0" encoding="' . $encoding . '" ?>' . LINEFEED;
263. if (!HIDE_ADVERTISE)
264. $this->m_out.="<!-- Generated by RdfSerializer.php from RDF RAP.".LINEFEED."# http://www.wiwiss.fu-berlin.de/suhl/bizer/rdfapi/index.html !-->".LINEFEED.LINEFEED ;

266. // write entitie declarations
267. if($this->use_entities) {
268. $this->m_out .= '<!DOCTYPE ' . $this->rdf_qname_prefix .
269. RDF_RDF.' [' . LINEFEED;
270. $this->writeEntityDeclarations();
271. $this->m_out .= LINEFEED . ']>' . LINEFEED;
272. }

274. // start the RDF text
275. $this->m_out .= '<' . $this->rdf_qname_prefix . RDF_RDF;

277. // write the xml:base
278. if ($model->getBaseURI() != NULL)
279. $this->m_out .= LINEFEED. INDENTATION .'xml:base="'.$model->getBaseURI().'"';

281. // write namespaces declarations
282. $this->writeNamespaceDeclarations();
283. $this->m_out .='>'. LINEFEED;

285. // write triples
286. $this->writeDescriptions();

288. $this->m_out .= LINEFEED;
289. $this->m_out .='</' . $this->rdf_qname_prefix . RDF_RDF .'>';

291. $this->m_namespaces=null;
292. $this->m_statements=null;
293. $this->m_currentSubject=null;
294. $this->m_groupTypeStatement=null;
295. $this->m_attributeStatements=null;
296. $this->m_contentStatements=null;
297. $this->m_rdfResourceElementText=null;

299. return $this->m_out;
300. }

302. /**
303. * Serializes a model and saves it into a file.
304. * Returns FALSE if the model couldn't be saved to the file.
305. *
306. * @param     object MemModel $model
307. * @param     String $encoding
308. * @return    boolean
309. * @access    public
310. */
311. function  saveAs(&$model, $filename, $encoding = DEFAULT_ENCODING) {
312. // serialize model
313. $RDF = $this->serialize($model, NULL, $encoding);

315. //write serialized model to file
316. $file_handle = @fopen($filename, 'w');
317. if ($file_handle) {
318. fwrite($file_handle, $RDF);
319. fclose($file_handle);
320. return TRUE;
321. } else {
322. return FALSE;
323. };
324. }

326. /**
327. * @access   private
328. */
329. function writeEntityDeclarations() {
330. foreach($this->m_namespaces as $prefix => $namespace) {
331. $this->m_out .= INDENTATION . '<!ENTITY '. $prefix . " '" . $namespace ."'>".LINEFEED;
332. }
333. }

335. /**
336. * @access   private
337. */
338. function writeNamespaceDeclarations() {
339. foreach($this->m_namespaces as $prefix => $namespace) {

341. if ($prefix == RDF_NAMESPACE_PREFIX && !$this->rdf_qnames) {

343. if($this->use_entities) {
344. $this->m_out .= LINEFEED . INDENTATION .XML_NAMESPACE_DECLARATION_PREFIX .
345. '="&' . $prefix . ';"';
346. } else {
347. $this->m_out .= LINEFEED . INDENTATION .XML_NAMESPACE_DECLARATION_PREFIX .
348. '="' . $namespace . '"';
349. }
350. } else {
351. if ($prefix == NULL)
352. $colon_prefix = $prefix;
353. else
354. $colon_prefix = ":" .$prefix;

356. if($this->use_entities) {
357. $this->m_out .= LINEFEED . INDENTATION .XML_NAMESPACE_DECLARATION_PREFIX
358. .$colon_prefix .'="&' . $prefix . ';"';
359. } else {
360. $this->m_out .= LINEFEED . INDENTATION .XML_NAMESPACE_DECLARATION_PREFIX
361. .$colon_prefix .'="' . $namespace . '"';
362. }
363. }
364. }
365. }

367. /**
368. * @access   private
369. */
370. function writeDescriptions()  {

372. $this->m_groupTypeStatement = NULL;
373. $this->m_attributeStatements = array();
374. $this->m_contentStatements = array();
375. $this->m_currentSubject = NULL;

377. foreach($this->m_statements as $key => $statement) {
378. $subject = $statement->getSubject();
379. $predicate = $statement->getPredicate();
380. $object = $statement->getobject();

382. // write Group and update current subject if nessesary
383. if ($this->m_currentSubject==NULL || !$this->m_currentSubject->equals($subject)) {
384. $this->writeGroup();
385. $this->m_currentSubject=$subject;
386. }

388. // classify the statement
389. if (($predicate->getURI() == RDF_NAMESPACE_URI.RDF_TYPE) && is_a($object, 'Resource')) {
390. if ($this->m_groupTypeStatement)
391. $this->writeGroup();
392. $this->m_groupTypeStatement = $statement;
393. }
394. elseif ($this->canAbbreviateValue($object) &&
395. $this->use_attributes &&
396. $this->checkForDoubleAttributes($predicate))
397. {
398. if (is_a($object, 'Literal')) {
399. if ($object->getDatatype() == NULL) {
400. $this->m_attributeStatements[] = $statement;
401. } else {
402. $this->m_contentStatements[] = $statement;
403. }
404. } else {
405. $this->m_attributeStatements[] = $statement;
406. }
407. } else {
408. $this->m_contentStatements[] = $statement;
409. }
410. }
411. $this->writeGroup();
412. }

414. /**
415. * @access   private
416. */
417. function writeGroup() {
418. if ($this->m_currentSubject==NULL || ($this->m_groupTypeStatement==NULL && (count($this->m_attributeStatements)==0) && (count($this->m_contentStatements)==0)))
419. return;
420. if ($this->m_groupTypeStatement!=NULL)
421. $outerElementName=$this->getElementText($this->m_groupTypeStatement->obj->getURI());
422. else
423. $outerElementName = $this->rdf_qname_prefix . RDF_DESCRIPTION;
424. $this->m_out .= LINEFEED . '<';
425. $this->m_out .= $outerElementName;

427. $this->m_out .= ' ';


430. $this->writeSubjectURI($this->m_currentSubject);

432. // attribute Statements
433. if ($this->use_attributes)
434. $this->writeAttributeStatements();

436. if (count($this->m_contentStatements)==0)
437. $this->m_out .= '/>' . LINEFEED;
438. else {
439. $this->m_out .= '>' . LINEFEED;

441. // content statements
442. $this->writeContentStatements();

444. $this->m_out .= '</';
445. $this->m_out .= $outerElementName;
446. $this->m_out .= '>'. LINEFEED;
447. }
448. $this->m_groupTypeStatement = NULL;
449. $this->m_attributeStatements = array();
450. $this->m_contentStatements = array();
451. }

453. /**
454. * @param object Node $predicate
455. * @access   private
456. */
457. function checkForDoubleAttributes($predicate) {
458. foreach($this->m_attributeStatements as $key => $statement) {
459. if ($statement->pred->equals($predicate))
460. return FALSE;
461. }
462. return TRUE;
463. }

465. /**
466. * @param STRING $uri
467. * @access   private
468. */
469. function relativizeURI($uri) {
470. $uri_namespace = RDFUtil::guessNamespace($uri);
471. if ($uri_namespace == $this->m_baseURI) {
472. return RDFUtil::guessName($uri);
473. } else {
474. return $uri;
475. }
476. }

478. /**
479. * @param object Node $subject_node
480. *
481. * @access   private
482. */

484. function writeSubjectURI($subject_node) {
485. $currentSubjectURI = $subject_node->getURI();
486. $relativizedURI = $this->relativizeURI($currentSubjectURI);

488. // if submitted subject ist a blank node, use rdf:nodeID
489. if (is_a($this->m_currentSubject, 'BlankNode')) {
490. $this->m_out .= $this->rdf_qname_prefix . RDF_NODEID;
491. $this->m_out .= '="';
492. $this->m_out .= $relativizedURI;
493. } else {


496. if (!($relativizedURI == $currentSubjectURI)) {
497. $this->m_out .= $this->rdf_qname_prefix . RDF_ID;
498. $this->m_out .= '="';
499. $this->m_out .= $relativizedURI;
500. } else {
501. $this->m_out .= $this->rdf_qname_prefix . RDF_ABOUT;
502. $this->m_out .= '="';
503. $this->writeAbsoluteResourceReference($relativizedURI);
504. };
505. };
506. $this->m_out .= '"';
507. }

509. /**
510. * @access   private
511. */
512. function writeAttributeStatements() {
513. foreach($this->m_attributeStatements as $key => $statement) {
514. $this->m_out .= LINEFEED;
515. $this->m_out .= INDENTATION;
516. $this->m_out .= $this->getElementText($statement->pred->getURI());
517. $this->m_out .= '=';
518. $value=$statement->obj->getLabel();
519. $quote=$this->getValueQuoteType($value);
520. $this->m_out .= $quote;
521. $this->m_out .= $value;
522. $this->m_out .= $quote;
523. }
524. }

526. /**
527. * @access   private
528. */
529. function writeContentStatements()  {
530. foreach($this->m_contentStatements as $key => $statement) {
531. $this->m_out .= INDENTATION;
532. $this->m_out .= '<';
533. $predicateElementText=$this->getElementText($statement->pred->getURI());
534. $this->m_out .= $predicateElementText;

536. if (is_a($statement->obj, 'Resource')) {
537. $this->writeResourceReference($statement->obj);
538. $this->m_out .= '/>' . LINEFEED;
539. } else {
540. if(is_a($statement->obj, 'Literal')) {
541. if ($statement->obj->getDatatype()!= NULL)
542. if ($statement->obj->getDatatype()== RDF_NAMESPACE_URI . RDF_XMLLITERAL) {
543. $this->m_out .= ' ' . RDF_NAMESPACE_PREFIX . ':' . RDF_PARSE_TYPE . '="' . RDF_PARSE_TYPE_LITERAL . '"';
544. } else {
545. $this->m_out .= ' ' . RDF_NAMESPACE_PREFIX . ':' . RDF_DATATYPE . '="' . $statement->obj->getDatatype() . '"';
546. }
547. if ($statement->obj->getLanguage()!= NULL)
548. $this->m_out .= ' ' . XML_NAMESPACE_PREFIX . ':' . XML_LANG . '="' . $statement->obj->getLanguage() . '"';
549. };
550. $this->m_out .= '>';
551. if ($statement->obj->getDatatype()== RDF_NAMESPACE_URI . RDF_XMLLITERAL) {
552. $this->m_out .= $statement->obj->getLabel();
553. } else {
554. $this->writeTextValue($statement->obj->getLabel());
555. }
556. $this->m_out .= '</';
557. $this->m_out .= $predicateElementText;
558. $this->m_out .= '>' . LINEFEED;
559. }
560. }
561. }

563. /**
564. * @param Object $object_node
565. * @access   private
566. */
567. function writeResourceReference($object_node)  {
568. $rebaseURI = $object_node->getURI();
569. $this->m_out .= ' ';
570. if (is_a($object_node, 'BlankNode')) {
571. $this->m_out .= $this->rdf_qname_prefix . RDF_NODEID;
572. } else {
573. $this->m_out .= $this->rdf_qname_prefix . RDF_RESOURCE;
574. };

576. $this->m_out .= '="';
577. $relativizedURI = $this->relativizeURI($rebaseURI);
578. if (!($relativizedURI == $rebaseURI))
579. if (!is_a($object_node, 'BlankNode'))
580. $this->m_out .= '#' . $relativizedURI;
581. else
582. $this->m_out .=  $relativizedURI;
583. else
584. $this->writeAbsoluteResourceReference($rebaseURI);
585. $this->m_out .= '"';
586. }


589. /**
590. * @param String $rebaseURI
591. * @access   private
592. */
593. function writeAbsoluteResourceReference($rebaseURI) {
594. $namespace=RDFUtil::guessNamespace($rebaseURI);
595. $localName=RDFUtil::guessName($rebaseURI);
596. $text=$rebaseURI;
597. if ($namespace!='' and $this->use_entities) {
598. $prefix= array_search($namespace, $this->m_namespaces);
599. $text='&'.$prefix.';'.$localName;
600. } else $text = RDFUtil::escapeValue($text);
601. $this->m_out .= $text;
602. }

604. /**
605. * @param STRING $textValue
606. * @access   private
607. */
608. function writeTextValue($textValue) {
609. if ($this->getValueQuoteType($textValue)==USE_CDATA)
610. $this->writeEscapedCDATA($textValue);
611. else
612. $this->m_out .= $textValue;
613. }

615. /**
616. * @param STRING $textValue
617. * @access   private
618. */
619. function writeEscapedCDATA($textValue) {
620. $this->m_out .= '<![CDATA[' . $textValue . ']]>';
621. }

623. /**
624. * @param STRING $textValue
625. * @access   private
626. */
627. function getValueQuoteType($textValue) {
628. $quote=USE_ANY_QUOTE;
629. $hasBreaks=FALSE;
630. $whiteSpaceOnly=TRUE;
631. for ($i=0; $i<strlen($textValue); $i++) {
632. $c=$textValue{$i};
633. if ($c=='<' || $c=='>' || $c=='&')
634. return USE_CDATA;
635. if ($c==LINEFEED)
636. $hasBreaks=TRUE;
637. if ($c=='"' || $c=="\'") {
638. if ($quote==USE_ANY_QUOTE)
639. $quote=($c=='"') ? "\'" : "\"";
640. elseif ($c==$quote)
641. return USE_CDATA;
642. }
643. if (!($c == ' '))
644. $whiteSpaceOnly = FALSE;
645. }
646. if ($whiteSpaceOnly || $hasBreaks)
647. return USE_CDATA;
648. return $quote==USE_ANY_QUOTE ? '"' : $quote;
649. }

651. /**
652. * @param object Node $node
653. * @access   private
654. */
655. function canAbbreviateValue($node) {
656. if (is_a($node, 'Literal')) {
657. $value= $node->getLabel();
658. if (strlen($value)<MAX_ALLOWED_ABBREVIATED_LENGTH) {
659. $c=$this->getValueQuoteType($value);
660. return $c=='"' || $c=='\'';
661. }
662. }
663. return FALSE;
664. }

666. /**
667. * @param STRING $elementName
668. * @access   private
669. */
670. function getElementText($elementName)  {
671. $namespace=RDFUtil::guessNamespace($elementName);
672. $localName=RDFUtil::guessName($elementName);
673. if ($namespace=="")
674. return $localName;
675. $prefix=array_search($namespace, $this->m_namespaces);

677. if ($prefix===FALSE) {
678. $errmsg = RDFAPI_ERROR . "(class: Serializer; method: getElementText): Prefix for element '" . $elementName . "' cannot be found.";
679. trigger_error($errmsg, E_USER_ERROR);
680. }
681. switch ($prefix) {
682. case RDF_NAMESPACE_PREFIX:
683. return $this->rdf_qname_prefix . $localName;
684. case NULL:
685. return $localName;
686. default:
687. return $prefix. ":" .$localName;
688. }
689. }

691. /**
692. * @param object MemModel $model
693. * @access   private
694. */
695. function collectNamespaces($model)  {
696. foreach($model->triples as $key => $value) {

698. if ($this->use_entities) {
699. $this->collectNamespace($value->getSubject());
700. if(!is_a($value->getObject(), 'Literal'))
701. $this->collectNamespace($value->getObject());

703. } else {

705. if  ($value->pred->getURI() == RDF_NAMESPACE_URI.RDF_TYPE)
706. $this->collectNamespace($value->getObject());
707. elseif
708. (($value->pred->getURI() == RDF_NAMESPACE_URI.RDFS_SUBCLASSOF) ||
709. ($value->pred->getURI() == RDF_NAMESPACE_URI.RDFS_SUBPROPERTYOF)) {
710. $this->collectNamespace($value->getSubject());
711. $this->collectNamespace($value->getObject());
712. }
713. }

715. $this->collectNamespace($value->getPredicate());

717. }
718. }

720. /**
721. * @param object Resource $resource
722. * @access   private
723. */
724. function collectNamespace($resource)  {

726. $namespace=RDFUtil::getNamespace($resource);
727. if (!in_array($namespace, $this->m_namespaces)&&$namespace!='') {
728. $prefix = array_search( $namespace, $this->m_defaultNamespaces);
729. if ($prefix===FALSE)
730. $prefix=$this->getNextNamespacePrefix();
731. $this->m_namespaces[$prefix] = $namespace;
732. }
733. }

735. /**
736. * @access   private
737. */
738. function getNextNamespacePrefix() {
739. $this->m_nextAutomaticPrefixIndex++;
740. return GENERAL_PREFIX_BASE . $this->m_nextAutomaticPrefixIndex;
741. }

743. }
744. ?>
```

Documentation generated on Thu, 7 Jul 2005 13:43:03 +0200 by phpDocumentor 1.3.0RC3
